# Porcine Protein Hydrolysates (PEPTEIVA^®^) Promote Growth and Enhance Systemic Immunity in Gilthead Sea Bream (*Sparus aurata*)

**DOI:** 10.3390/ani11072122

**Published:** 2021-07-16

**Authors:** Enric Gisbert, Antoni Ibarz, Joana P. Firmino, Laura Fernández-Alacid, Ricardo Salomón, Eva Vallejos-Vidal, Alberto Ruiz, Javier Polo, Ignasi Sanahuja, Felipe E. Reyes-López, Lluis Tort, Karl B. Andree

**Affiliations:** 1 IRTA, Centre de Sant Carles de la Ràpita, Aquaculture Program, Carretera Poble Nou, km 5.5, 43540 Sant Carles de la Ràpita, Spain; joana.firmino@irta.cat (J.P.F.); ricardo.salomon@irta.es (R.S.); alberto.ruiz@irta.cat (A.R.); karl.andree@irta.cat (K.B.A.); 2 Department of Cell Biology, Physiology and Immunology, Faculty of Biology, University of Barcelona, Avda. Diagonal 643, 08028 Barcelona, Spain; tibarz@ub.edu (A.I.); fernandez_alacid@ub.edu (L.F.-A.); isanahuja@ub.edu (I.S.); 3 Centro de Biotecnología Acuícola, Departamento de Biología, Facultad de Química y Biología, Universidad de Santiago de Chile, Santiago 9170002, Chile; eva.vallejos@gmail.com (E.V.-V.); Felipe.Reyes@uab.cat (F.E.R.-L.); 4 APC Europe SL, Avda. Sant Julià 246-258, 08403 Granollers, Spain; javier.polo@apc-europe.com; 5 Department of Cell Biology, Physiology and Immunology, Universitat Autònoma de Barcelona, 08193 Bellaterra, Spain; Lluis.Tort@uab.cat; 6 Facultad de Medicina Veterinaria y Agronomía, Universidad de Las Américas, Providencia, Santiago 7500975, Chile; 7 Consorcio Tecnológico de Sanidad Acuícola, Ictio Biotechnologies S.A., Santiago 7500000, Chile

**Keywords:** aquaculture, functional feed, protein hydrolysates, low fishmeal diet, pathogen-associated molecular pattern (PAMP), skin-associated lymphoid tissue (SALT)

## Abstract

**Simple Summary:**

The development of functional feeds based on additives intended for supporting somatic growth, as well as promoting and modulating the host’s immune response is a promising and reliable strategy in the post-antibiotic era. In this study, we have evaluated porcine plasma protein hydrolysate (PPH), a by-product of the rendering industry, as a functional ingredient in aquafeeds. Thus, a 92-day nutritional trial was conducted to evaluate the inclusion of PPH in gilthead sea bream (*Sparus aurata*) diets. In particular, the control diet contained 7% fishmeal (48% protein, 17% fat, and 22 MJ kg^−1^ gross energy), whereas the PPH was included in the experimental diet at the expense of 5% fish meal. Results indicated that this rendering by-product had a beneficial effect on the growth performance and feed-efficiency parameters, as well as promoted systemic immunity. In addition, no differences in biochemical skin mucus biomarkers were found between both groups. The present study indicated that porcine protein hydrolysate obtained from blood plasma may be considered as a safe and functional ingredient for aquafeeds.

**Abstract:**

The effects of porcine plasma protein hydrolysate (PPH) on growth, feed efficiency, and immune responses was evaluated in *Sparus aurata*. Fish were fed two isoproteic (48% protein), isolipidic (17% fat), and isoenergetic diets (21.7 MJ/kg) diets, one of them containing 5% PPH at the expense of fishmeal. Both diets were tested for 92 days. A significant increase in growth was observed in fish fed the PPH diet in comparison to the control group (182.2 ± 4.4 vs. 173.8 ± 4.1 g), as well as an increase in feed intake without worsening FCR values. An ex vivo assay, with splenocytes incubated with lipopolysaccharide, was conducted to evaluate the cellular immune competence of fish. Genes involved in humoral immunity *(lys*, *IgM*), pro- (*tnf-α*, *il-1β*), and anti-inflammatory (*tgf-β1*, *il10*) cytokines were upregulated in the PPH group in comparison to the control group. The inclusion of PPH in diets enhanced the antibacterial capacity of skin mucus, as the co-culture of selected bacteria *(E. coli*, *V. anguillarum*, and *P. anguilliseptica*) with skin mucus indicated. The present results showed that the PPH in low fishmeal diets (2%) promoted growth and feed efficiency, as well as enhancing the immune response, which indicates that this is a safe and functional ingredient for aquafeeds.

## 1. Introduction

Despite the considerable progress in the aquaculture sector in enhancing its efficiency in the use of marine resources over the past 20 years, aquafeeds are still dependent on capture fisheries, although to a lesser extent, due to improvement in the feed conversion rates, reduced fishmeal, and fish oil inclusion ratios, as well as the increased use of fishmeal from trimmings [1]. Regardless of these remarkable advances, fish protein hydrolysates are still used at moderate inclusion rates in most larval and fingerling diets, as well as in grow-out, broodstock, and finishing feeds. This general use is based on the fact that fish protein hydrolysates are valuable sources of proteins, amino acids, peptides, and antioxidants, whose inclusion in aquafeeds has the potential to improve growth and feed utilization, modulate immune responses, and enhance disease resistance in fish [2]. Along these lines, fish diseases are considered a main persistent threat to aquaculture, which represents an annual estimated loss of US$6 billion at a global scale [3]. Although the use of antibiotics has been reduced in aquaculture, the indiscriminate prophylactic use of antibiotics associated with intensive aquaculture practices can still be observed among some of the major aquaculture-producing countries [4,5]. Nevertheless, several countries, including the EU, will prohibit all forms of routine antibiotic use in farming in 2022, which highlights the importance for the development of more sustainable alternative preventive treatments [6]. Under this scenario, novel policies and sustainable production systems addressing health management and animal welfare issues are mandatory [7].

The development of functional feeds based on promoting and modulating the host’s immune response is a promising and reliable strategy [8,9,10,11]. Among the vast list of functional feed additives for promoting fish health [12], the inclusion of protein hydrolysates in aquafeeds is of interest beyond their nutritional value, since they are reputed for their antimicrobial, antioxidant, and immunomodulatory properties [13,14,15,16]. Although fish protein hydrolysates have been extensively studied in different fish species and at different stages of the production cycle [2], the potential use of other sources of animal and plant protein hydrolysates have not been extensively evaluated in fish [17], regardless of their promising properties as functional ingredients [15]. In animal production, high-quality proteins are generally not used as a source of feed additives, since they may have other final uses (i.e., meals); thus, animal by-products are the main ingredients hydrolyzed to produce peptides for animal feeds [2]. Among the different sources of raw materials to be used for producing protein hydrolysates, rendering by-products have been reported to have relevant nutritional and functional roles in fish nutrition [12,18,19,20]. In particular, while porcine spray-dried plasma is the most common evaluated blood by-product in aquafeeds [19,20,21,22], blood protein hydrolysates may yet be an untapped safe source of animal protein hydrolysates for aquafeeds [23]. In addition, the use of porcine blood derivatives has environmental benefits for the sustainability and circular economy associated with animal production because it helps to provide added value to the industry and has a lower carbon footprint compared with vegetable proteins.

In the present study, the authors aimed to evaluate the effects of porcine protein hydrolysates (PPH; commercial name: PEPTEIVA^®^, APC Europe SL, Barcelona, Spain) on the most common key performance indicators, such as the growth and feed-efficiency parameters, as well as on its influence on gilthead sea bream (*Sparus aurata*) immunity. This species is recognized as the most important Mediterranean aquaculture fish species in terms of volume and economic value [24]. Thus, the immunomodulatory response of the dietary administration of PPH to a bacterial challenge was tested at two levels: (i) by evaluating the level of gene expression in splenocytes exposed to short-term ex vivo stimulation with lipopolysaccharide (LPS); and (ii) by measuring the antibacterial activity of the skin mucus when incubated with different bacterial strains.

## 2. Materials and Methods

### 2.1. Fish, Diets, and Sampling

Gilthead sea bream juveniles (body weight, BW = 5–8 g) were obtained from a commercial fish farm (PISCIMAR SL, Andromeda Group, Burriana, Spain). After acclimation of the fish (21 days), they were individually measured in BW (g) and standard length (SL, cm), then homogeneously distributed in eight 450 L tanks (35 fish per tank; N = 280) under an open-flow water regime (June–September; latitude 40°37′41″ N). Fish were fed two experimental diets (4 replicates per diet) for 92 days under the following environmental conditions: water temperature values ranging from 22 to 27 °C (23.1 ± 1.1 °C; mean ± standard deviation), 6.1 ± 0.2 mg L^−1^ of dissolved oxygen (OXI330, Crison Instruments, Barcelona, Spain), 35.1 ± 0.1 ppt of salinity (MASTER-20 T; ATAGO Co. Ltd., Tokyo, Japan), 7.5 ± 0.01 pH (pH meter 507, Crison Instruments; Barcelona, Spain), and a natural photoperiod. Feeds were distributed four times per day by automatic feeders (ARVO-TEC T Drum 2000^TM^, Arvotec, Finland), at the initial feeding rate of 3.3% of the stocked biomass, which approached apparent satiation. The feeding rate was regularly adjusted depending on the amount of uneaten feed pellets recovered from the bottom of the tank from the previous day in order to guarantee that feed was offered in excess. For this purpose, one hour after feed administration, the uneaten pellets were recovered from the bottom of the tank, dried in an oven (120 °C), and their dry weight used for estimating the amount of uneaten feed and for calculating the feed intake.

The control diet was formulated with low levels of fishmeal (FM) (7% FM) to contain 48% crude protein, 17% crude fat, and 22 MJ kg^−1^ gross energy, meeting the nutritional requirements of gilthead sea bream juveniles. Based on this basal formulation, an experimental diet was formulated, in which porcine protein hydrolysate (PPH) (PEPTEIVA^®^; APC Europe, SA, Granollers, Spain) was incorporated at 5% at the expense of FM (final FM levels = 2%). The PPH is a hydrolysate of porcine plasma containing 76% protein (>85% of protein in form of peptides smaller than 10 KDa), 2.6% crude fat, and 14.5% ash. Both diets were isonitrogenous, isolipidic, and isoenergetic (Table 1). Diets were manufactured by temperature-controlled extrusion by Sparos Lda. (Olhão, Portugal), as described in Gisbert et al. [19].

Fish growth in BW was measured every month in order to evaluate growth performance and adjust the feeding ratio to the stocked biomass. For that purpose, all fish in each tank were gently anesthetized (150 mg MS-222 L^−1^), and BW and SL were individually measured to the nearest 0.1 g and 1 mm, respectively. Fish growth was evaluated by means of the following indices: Fulton’s condition factor (K) = (BW_f_/SL_f_^3^) × 100; specific growth rate in BW (SGR_BW_, %) = ((ln BW_f_ − ln BW_i_) × 100)/time (d), where BW_f_ and BW_i_ correspond to final and initial BW, and SL_f_ corresponds to final SL, respectively. Feed utilization was evaluated by the following formula: feed conversion ratio (FCR) = feed intake (g)/increase of fish biomass (g).

### 2.2. Skin Mucus Collection, Biomarker Analyses, and Antibacterial Activity Measurement

At the end of the trial, 20 animals per diet (5 specimens per tank) were randomly collected from each experimental diet and slightly anaesthetized as previously described and mucus collected as described in Fernández-Alacid et al. [24]. Briefly, skin mucus was collected in a very fast process (<2 min) using sterile glass slides from the over-lateral line in a front to caudal direction, and the skin mucus was carefully pushed and collected in a sterile tube (2 mL), avoiding the contamination with blood and/or urine-genital and intestinal excretions. Mucus samples were homogenized using a sterile Teflon implement to desegregate the mucus mesh before centrifugation at 14,000 × *g* for 15 min. The resultant mucus supernatants were collected, avoiding the surface lipid layer, aliquoted, and stored at −80 °C for further analyses.

The soluble protein concentration in skin mucus (mg of protein mL^−1^) was measured by means of the Bradford assay [25], using bovine serum albumin (Sigma-Aldrich, Madrid, Spain) as a standard. Glucose (μg mL^−1^) and lactate (μg mg^−1^) concentrations were determined by their respective enzymatic colorimetric tests (SPINREACT^®^, Barcelona, Spain) following the manufacturer’s instructions, but with slight modifications as described in Fernández-Alacid et al. [24]. Mucus cortisol levels (ng cortisol mL^−1^ of skin mucus and ng g^−1^ of mucus protein) were measured using an ELISA kit (IBL International, Germany), as was previously described for fish mucus samples [24,26]. Briefly, a volume of 50 μL of mucus extract or standard solutions were mixed with the enzyme conjugate (100 μL) and incubated for 2 h at room temperature (RT). The substrate solution (100 μL) was added after rinsing the wells with a wash solution, and incubated for 30 min. The reaction was stopped by adding 100 µL of stop solution and the OD read at λ = 450 nm. Ferric antioxidant status as a measurement of the serum’s antioxidant power was determined by an enzymatic colorimetric test (ferric antioxidant status detection kit, Invitrogen, Madrid, Spain). Following the manufacturer’s instructions for plasma determinations but with slight modifications, 20 μL of the mucus extract or standard solution (from 0 to 1000 μmol μL^−1^ of FeCl_2_) was mixed with 75 μL of the kit color solution and incubated for 30 min at RT. The OD was read at λ = 560 nm. Antioxidant values were expressed as nmol of Ferric-ion Reducing Antioxidant Power (FRAP) mL^−1^ of skin mucus, and nmol per mg of mucus protein^−1^. All measurements were conducted in triplicate (methodological replicates) with a microplate spectrophotometer reader (Infinity 171 Pro200 spectrophotometer, Tecan, Zurich, Switzerland).

The study of mucus antibacterial activity in gilthead sea bream juveniles fed experimental diets was performed as described in Sanahuja et al. [27] using three different bacteria: a non-pathogenic bacterium for fish, *Escherichia coli* (DSMZ423), and two pathogenic bacteria for marine fish species, *Vibrio anguillarum* (CECT522T) and *Pseudomonas anguilliseptica* (CECT899T). *E. coli* were grown in Tryptic Soy Broth culture media (TSB, Conda, Spain), whereas *V. anguillarum* and *P. anguilliseptica* were grown in Marine Broth culture media (MB, Difco Laboratories, Detroit, MI, USA). The effect of skin mucus on bacterial viability was determined by monitoring the absorbance of the bacterial cultures grown in flat-bottomed 96-well plates. In particular, each well was filled with 50 μL of bacterial suspension (OD = 0.2) in the appropriate culture media (1×) plus 100 μL of skin mucus (4 μg μL^−1^ of mucus protein) and 50 μL of culture media (3×) to obtain a 200 μL final volume. Putative bacterial growth of fish mucus origin was measured by adding 100 μL of culture media (2×) and 100 μL of skin mucus (2 μg μL^−1^ of mucus protein) without bacterial suspension. Additionally, bacterial growth without mucus (control values) were prepared by adding 50 μL of bacterial suspension (OD = 0.2) and 150 μL of culture media (1×). Blanks (control bacterial growth without bacteria and mucus) were prepared by adding 200 μL culture media (1×). The absorbance of the bacteria, control values, and blanks were measured at λ = 400 nm every 30 min for 14 h at 25 °C in flat-bottomed 96-well plates. In each time point, the average absorbance of the controls without bacteria (skin mucus at 2 μg μL^−1^ of protein (100 μL) plus 100 μL of medium) was subtracted from the absorbance from co-culture (bacteria plus skin mucus) samples. All assays were done in triplicate (methodological replicates). Data are presented as growth curves (increased absorbance at λ = 400 nm per unit of time) and as percentage of inhibition with respect to bacterial growth for each two hours of co-culture with skin mucus.

### 2.3. Ex Vivo Immune Stimulation of Splenocytes with LPS and Gene Expression Analysis

At the end of the nutritional trial, 6 specimens from each experimental group (biological replicates) were sacrificed with an overdose of anesthetic, and their spleens removed. The ex vivo protocol was similar to that described by Salomón et al. [28]. In brief, the spleen of each fish was passed through a 100 mm nylon mesh cell strainer (SefarNytal PA-13xxx/100, Barcelona, Spain) in Leibovitz L15 medium (Gibco^TM^, Thermo Fisher Scientific, Waltham, MA, USA) containing a mixture of penicillin and streptomycin (10,000 IU mL^−1^; Gibco^TM^) at 1:1000 and 2% fetal calf serum (Gibco^TM^). The resulting cell suspension was collected and centrifuged at 400× *g* for 10 min at RT. Then, the supernatant was discarded and replaced with 10 mL of Leibovitz L15 medium. The cell suspension was again centrifuged, the supernatants removed, and replaced with 30 mL of media. Cells were distributed into 12-well microtiter plates in 5 mL aliquots (2 wells per fish). In order to evaluate the immune response of the spleen cells to a bacterial-type PAMP, LPS (Sigma) at a dose of 50 mg mL^−1^ was added to each well of the microtiter plate (Greiner Bio-One España, Madrid, Spain). Control samples included 250 mL of PBS. In order to evaluate the immune response of the cultured cells, splenocytes were regularly harvested at 4, 12, and 24 h after LPS stimulation, centrifuged at 400× *g* for 10 min at RT, and the supernatant discarded. The sampling point of 0 h was considered before the addition of the LPS into cell cultures. After cell centrifugation, the pellet was suspended in RNAlater^®^ (Sigma-Aldrich, Madrid, Spain).

Total RNA from the splenocytes’ primary cell cultures (SPCC) harvested at 0, 4, 12, and 24 h of incubation was extracted using the QIAGEN RNeasy^®^ Mini Kit (QIAGEN, Hilden, Germany). The amount of total RNA was determined by spectrophotometry with an ND-2000 NanoDrop (Thermo Scientific™, Waltham, MA, USA) and its quality evaluated by means of agarose gel electrophoresis (2%). First-strand cDNA was synthesized in order to quantify the expression of the genes under study. For cDNA synthesis, 1 μg of total RNA was reverse transcribed using a high capacity cDNA reverse transcription kit (QuantiTect^®^ Reverse Transcription Kit, QIAGEN, Hilden, Germany) in a final reaction volume of 20 μL. The SPCC from gilthead sea bream fed the control and PPH diets and treated with PBS and LPS were analyzed by qRT-PCR in order to evaluate the expression of immune-related genes. This analysis included humoral innate response effectors (lysozyme (*lys*), immunoglobulin M (*igM*)), pro- (*il-1β, tnfα*) and anti-inflammatory cytokines (*il-10*, *tgfβ1*), the surface cell marker cd4 (*cd4*), and antioxidant enzyme genes (manganese superoxide dismutase (*mn-s*od) and catalase (*cat*)). β-actin (*β-act*) was used as a reference gene for relative gene quantification analysis. The specific primer sets for each gene are detailed in Salomón et al. [28].

The primer amplification efficiency for all the genes included in this analysis was determined using a serially diluted reference pool containing 1 μL of each sample. The primer efficiency was calculated according to the formula described in Pfaffl [29], which is based on the slope value obtained from the linear regression of the plotted Ct values from each serial dilution. Real-time PCR reactions were performed with a 2.5 μL iTaq universal Sybr green supermix (Bio-Rad Laboratories, Alcobendas, Spain), 0.1 μL forward and reverse primers (final concentration of 500 nM at the reaction volume), and 1.3 μL of miliQ H_2_O using a 1:4 cDNA dilution from all the cDNA stock samples. The thermal conditions used were 3 min at 95 °C of pre-incubation followed by 40 cycles at 95 °C for 30 s and 60 °C for 30 s. An additional temperature ramping step from 65 to 95 °C was included to obtain the melting curves and thus verify the amplification of a unique single product from all samples. All the reactions were performed in duplicate using a CFX384 Touch Real-Time PCR Detection System (Bio-Rad Laboratories, Alcobendas, Spain). Quantification was done according to Pfaffl’s method [29] corrected for the efficiency of each primer set obtained. The expression value for each experimental condition was expressed as the normalized relative expression (NRE), calculated in relation to the values of control group (SPCC at time zero) and normalized against those of the reference gene. The results were expressed as the mean expression values obtained at 0, 4, 12, and 24 h of incubation with PBS or LPS (*n* = 6 fish per diet, experimental condition, and time-point assessed). The expression value for each experimental condition was expressed as the normalized relative expression (NRE), calculated in relation to values of the control group (SPCC at time zero) and normalized against those of the reference gene, as previously described [28].

### 2.4. Statistical Analyses

All results are expressed as the mean ± standard deviation. All data were checked for normality and homoscedasticity prior to their analysis. Differences in somatic growth, FCR, and skin mucus biomarkers between both groups were evaluated by means of a t-test. Changes in gene expression of SPCC were analyzed by a two-way ANOVA considering both dietary groups and sampling times (0, 4, 12, and 24 h of incubation) as factors. When there were statistically significant differences among groups (*p* < 0.05), the ANOVA was followed by the post-hoc Bonferroni test. All statistical analyses were performed using GraphPad Prism V.6.1. (GraphPad Software, San Diego, CA, USA).

## 3. Results

### 3.1. Growth, Body Condition, and Feed Performance Indicators

Data on the fish survival, growth, and feed performance indicators are summarized in Table 2. No differences in mortality were observed between both experimental groups, with the average survival rates ranging between 96.8 and 97.6% (*p* > 0.05). At the end of the 92-day trial, fish fed the PPH diet were 4.6% heavier than their congeners fed the control diet (*p* < 0.05; Table 2). Similarly, SGR values in terms of BW were higher in fish fed the PPH diet than in those from the control group (*p* < 0.05). In contrast, no differences were found in terms of SL nor the Fulton’s condition factor between both experimental groups (*p* > 0.05). Gilthead sea bream fed the PPH diet showed similar FCR values (*p* > 0.05, as well as higher FI values than those fish fed the control diet (*p* < 0.05).

### 3.2. Skin Mucus Biomarkers and Bactericidal Activity

The impact of experimental diets on skin mucus biomarkers is represented in Table 3. In particular, no statistically significant differences were found in the skin mucus between both experimental groups for glucose, lactate, cortisol, protein, and FRAP concentrations (*p* > 0.05). Similar results were found when data were expressed as ratios considering the amount of soluble protein in skin mucus (*p* > 0.05).

**Figure 1:**
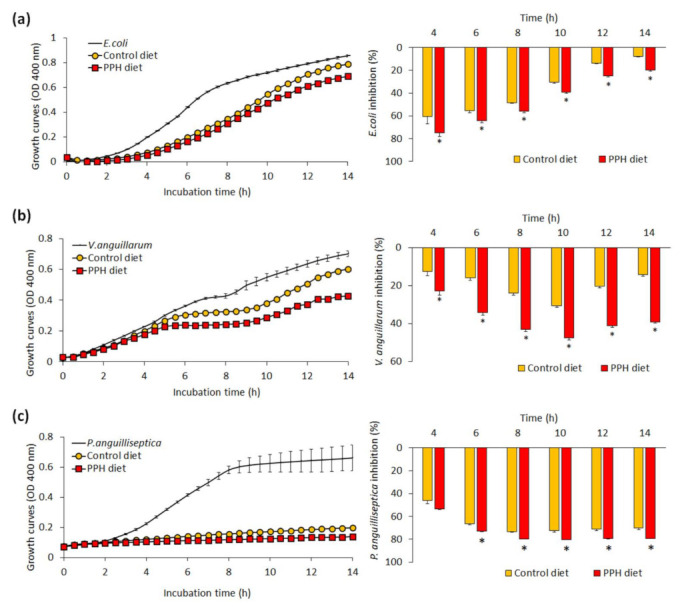
Bacterial growth (left) and inhibition rate (right) of *E. coli* (**a**), *V. anguillarum* (**b**), and *P. anguilliseptica* (**c**) co-cultured with gilthead sea bream (*Sparus aurata*) skin mucus from fish fed both experimental (control and PPH) diets. Data are shown as the mean ± standard error of the mean (*n* = 4 per dietary group). An asterisk (*) denotes statistically significant differences between both diets at particular sampling times (4, 6, 8, 10, 12, and 14 h) of the co-culture (*t*-test, *p* < 0.05).

The effects of dietary PPH on the bactericidal activity of skin mucus in gilthead sea bream juveniles is shown in Figure 1. In relation to the non-pathogenic bacteria *E. coli* co-cultured with gilthead sea bream skin mucus, its inhibitory capacity showed higher values from fish fed the PPH diet in comparison to the control group at any of the sampling times evaluated (4–14 h of incubation) (Figure 1a; *p* < 0.05). Similar results were found when co-culturing the skin mucus of gilthead sea bream from the PPH group with the pathogenic bacteria *V. anguillarum* (Figure 1b; *p* < 0.05). Regarding the inhibitory capacity of skin mucus when co-cultured with the pathogenic bacteria *P. anguilliseptica* no differences were found between both dietary groups at earlier co-incubation times (at 4 h) (*p* > 0.05), whereas higher inhibitory capacity was found between 6 and 14 h in the skin mucus of fish fed the PPH in relation to the control group (Figure 1c; *p* < 0.05).

### 3.3. Gene Expression Analysis of Splenocytes Stimulated by LPS in an Ex Vivo Assay

**Figure 2:**
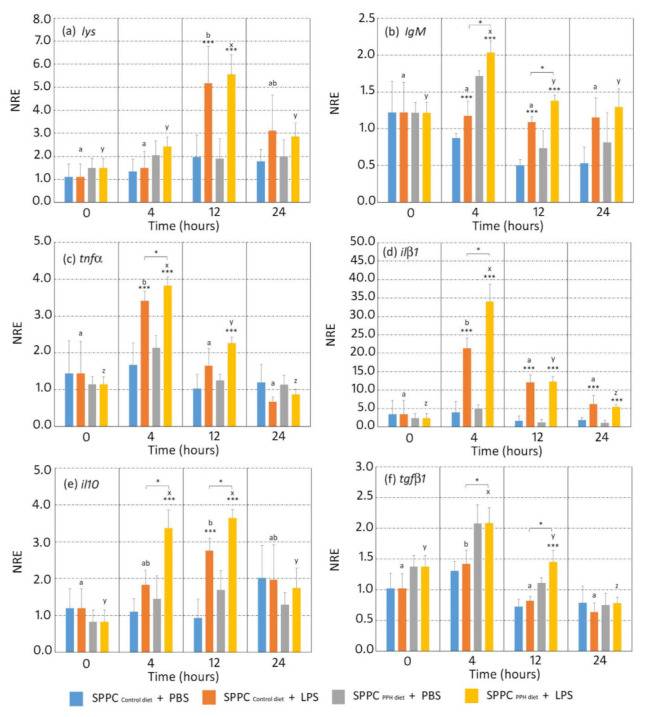
Normalized relative expression (NRE) of immune-related genes in gilthead sea bream (*Sparus aurata*) fed with experimental (control and PPH) diets. The expression of *lys* (**a**), *igM* (**b**), *tnf-α* (**c**), *il-1β* (**d**), *il-10* (**e**), and *tgf-β1* (**f**) were evaluated in splenocyte primary cell cultures (SPCC) isolated from gilthead sea breams at 4, 12, and 24 h after exposure to PBS or LPS. Orange and yellow bars: LPS-treated splenocytes from gilthead sea bream fed with control and PPH diets, respectively. Blue and grey bars: PBS-treated splenocytes from gilthead sea bream fed with the control and PPH diets, respectively. The time 0 h corresponds to the basal state prior to the beginning of the treatment. Data are shown as the mean ± standard error of the mean (*n* = 4 per dietary group). Statistical analysis: two-way ANOVA with Tukey’s post hoc test. An asterisk (*) represents significant differences between the dietary groups regarding the LPS treatment evaluated at the same time-point; (***) represents significant differences between splenocytes treated with PBS and LPS within the same diet and time point; different letters (a, b, and c) represent significant differences between the control diet different post-exposure times with LPS (*p* < 0.05). Different letters (x, y, and z) represent significant differences between the PPH diet at different post-exposure times with LPS (*p* < 0.05). Abbreviations: *igM*, immunoglobulin M; *il-1β*, interleukin 1 beta; *lys*, lysozyme; *tnf-α*, tumour necrosis factor alpha; *il-10*, interleukin 10; *tgf-β1*, transforming growth factor beta 1.

**Figure 3:**
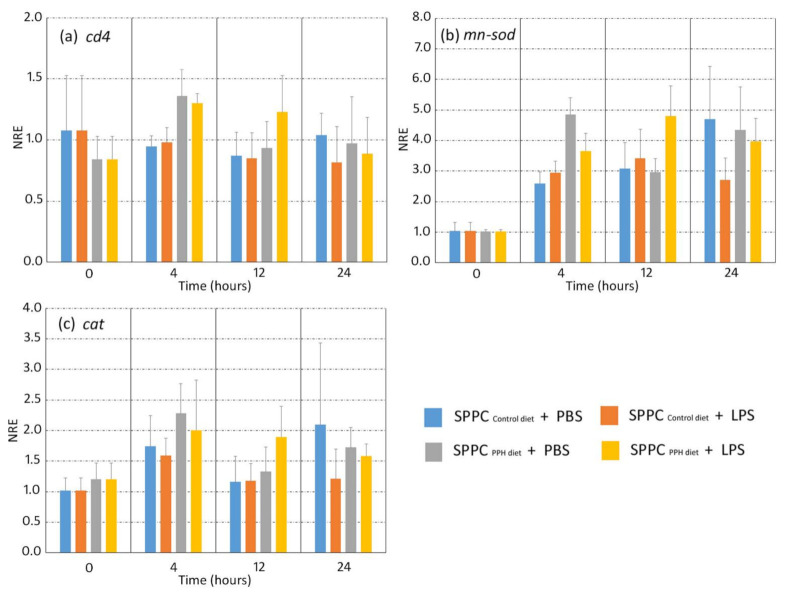
Normalized relative expression (NRE) of immune-related genes on gilthead sea bream (*Sparus aurata*) fed with experimental (control and PPH) diets. The expression of *cd4* (**a**), *mn-sod* (**b**), and *cat* (**c**) were evaluated in splenocyte primary cell cultures (SPCC) isolated from gilthead sea breams at 4, 12, and 24 h after exposure to PBS or LPS. Orange and yellow bars: LPS-treated splenocytes from gilthead sea bream fed with control and PPH diets, respectively. Blue and grey bars: PBS-treated splenocytes from gilthead sea bream fed with the control and PPH diets, respectively. The time 0 h corresponds to the basal state prior to the beginning of the treatment. Data are shown as the mean ± standard error of the mean (*n* = 4 per dietary group). No statistical differences were found between the dietary treatments and sampling points (two-way ANOVA, *p* > 0.05). Abbreviations: *cd4*, cluster of differentiation 4; *mn-sod*, manganese superoxide dismutase; *cat*, catalase.

Results in terms of gene expression of the selected gene markers involved in humoral immunity (*lys*, *IgM*), namely, the pro- (*tnf-α*, *il-1β*) and anti-inflammatory (*tgf-β1*, *il10*) cytokines and the leucocyte cell surface marker cd4, are shown in Figure 2, and the antioxidative stress enzymes (*mn-sod* and *cat*) are shown in Figure 3. When considering the expression of the selected genes involved in humoral immunity (*lys* and *igM*), both genes were modulated by the exposure to LPS. In particular, *lys* expression increased at 12 hpi in both experimental groups when splenocytes were incubated with LPS, although no differences in gene expression were found between them (*p* < 0.05). Gene expression values of *lys* were similar among both dietary groups and in splenocytes incubated with LPS and PBS at 4, 12, and 24 hpi (Figure 2a; *p* > 0.05). Regarding *igM*, no differences in expression were found in splenocytes from the control diet when exposed to LPS and PBS at 4 hpi (Figure 2a; *p* > 0.05). In contrast, *igM* expression was higher in splenocytes from fish fed the PPH diet when exposed to LPS in comparison to those from the same dietary group exposed to PBS (*p* > 0.05). At 12 hpi, *igM* expression values in splenocytes exposed to LPS from fish fed the PPH diet were higher than those of the same group exposed to PBS and to those from the control group exposed to LPS (Figure 2b; *p* < 0.05). At 24 hpi, no differences were found in the *igM* expression levels in splenocytes exposed to LPS between both dietary groups (*p* > 0.05), although values from the control group were higher than in splenocytes exposed to PBS (Figure 2b; *p* < 0.05).

Regarding the gene expression of pro-inflammatory cytokines, *tnf-α* in splenocytes exposed to LPS from both experimental groups increased at 4 hpi in comparison to splenocytes incubated with PBS, even though no differences in expression were found between the control and PPH groups (Figure 2c; *p* > 0.05). At 12 hpi, *tnf-α* expression was higher in splenocytes exposed to LPS from the PPH group in comparison to the control group (*p* < 0.05), whereas such differences disappeared at 24 hpi (Figure 2c; *p* > 0.05). Regarding *il-1*, higher expression values were found at 4 and 12 hpi in splenocytes incubated with LPS in both dietary groups in comparison to those incubated with PBS (*p* < 0.05), whereas expression values of *il-1β* were higher in the PPH group in comparison to the control group at 4 hpi (Figure 2d; *p* > 0.05). At 24 hpi, no differences in *il-1β* were found between both experimental groups, regardless of the splenocytes being incubated with PBS or LPS (Figure 2d; *p* > 0.05).

The expression of anti-inflammatory cytokines in splenocytes incubated with PBS and LPS was affected by the diet administered. In particular, expression values of *il10* from splenocytes incubated with LPS were higher than those incubated with PBS at 4 hpi (*p* < 0.05), whereas this expression was higher in splenocytes from the PPH group in comparison to the control group (Figure 2e; *p* < 0.05). At 12 hpi, *il10* expression values were higher in splenocytes incubated with LPS in comparison to those exposed to PBS in both dietary groups (*p* < 0.05), even though no differences were found between both groups (*p* > 0.05). At 24 hpi, expression values were similar between both dietary conditions, regardless of the splenocytes being incubated with PBS or LPS (Figure 2e; *p* > 0.05). Regarding *tgf-β1*, expression values at 4 and 12 hpi were higher in splenocytes incubated with LPS in both dietary groups in comparison to those incubated with PBS (*p* < 0.05). In addition, *tgf-β1* expression at 12 hpi was higher in splenocytes incubated with LPS in the PPH group in comparison to the control diet (Figure 2f; *p* < 0.05). At 24 hpi, no differences in *tgf-β1* were found between both dietary groups, regardless of splenocytes being incubated with PBS or LPS (Figure 2f; *p* > 0.05).

No differences in gene expression of the leucocyte cell surface marker *cd4* were found between the experimental groups at any of the sampling times (*p* < 0.05). No significant changes were found in terms of *mn-sod* and *cat* expression at 4, 12, and 24 hpi in both experimental groups when the splenocytes were exposed to LPS and PBS (Figure 3a–c; *p* > 0.05).

## 4. Discussion

Terrestrial and aquatic animal by-products have been progressively gaining acceptance and importance as aquafeed ingredients due to the short supply and high cost of FM. Generally, protein content in animal by-products is higher, and their levels of essential amino acids are superior to those of plant origin. Furthermore, they are also less expensive than FM, and thus very valuable for the formulation of cost-effective aquafeeds [2,23,30,31]. Moreover, their use as valued ingredients in animal diets promote the sustainability of the whole sector and contribute to the circular economy and environment because of having a lower carbon footprint than vegetal proteins [1]. Although recent changes in legislation allow the use of animal proteins of porcine and avian origin in aquafeeds [32,33], the use of rendering blood by-products remains an untapped source of high-quality ingredients for aquafeeds [23]. Under this new legislative scenario, studies evaluating the properties of these ingredients are needed in order to test their nutritional and functional properties as well as safe inclusion in aquafeeds.

Under current experimental conditions, the inclusion of PPH at 5% in diets with low FM levels (2%) exerted a positive effect on growth performance indicators such as BW_f_ and SGR_BW_. These results may be attributed to the high apparent digestibility values of porcine blood by-products derived from plasma [23,34,35,36], as well as their content in growth factors and biologically active peptides [37,38]. Similar results have been observed when evaluating other blood by-products in different fish species [19,20,21,22]. In the current study, the improvement in growth performance may be associated with higher FI values rather than changes to the results in FCR. In particular, the inclusion of PPH in diets with very low FM levels seemed to increase diet palatability, as feed intake increased in those animals when compared to the control group, results that may be attributed to the peptide and free amino acid profile of this ingredient [17]. These results have also been found in livestock [34] and fish [19,20,21] fed spray-dried plasma.

In addition to evaluating the potential growth-promoting effects of the terrestrial blood by-product evaluated in this study, the authors wanted to screen the potential immunomodulatory properties of PPH inclusion in diets with very low levels of FM by means of an ex vivo assay in which splenocytes from both dietary groups were stimulated with bacterial LPS. Results from this ex vivo assay revealed that PPH enhanced the immune response of gilthead sea bream. In particular, we found an upregulation of the gene markers involved in the humoral innate response (*igM*), as well as pro- (*il-1β*, *tnf-α*) and anti-inflammatory cytokines (*il-10*, *tgf-β1*).

Natural antibodies, such as immunoglobulins, are important actors in innate and, in particular, adaptive immunity, producing specific antibody responses against antigens. In this sense, IgM is the most abundant immunoglobulin in plasma and mucus in fish, and the key player in the orchestration of the systemic humoral immune response [39]. The higher *igM* expression in splenocytes from gilthead sea bream fed the PPH diet at 4 and 12 hpi when compared to that of the control group may indicate that the tested ingredient promoted the production of IgM, as shown by several studies testing feed additives with immunomodulatory properties [28,40,41]. The inflammatory response plays a key role in the control and removal of pathogens from the host. Under current experimental conditions, the ex vivo assay showed an early (4 hpi) upregulation of pro-inflammatory cytokines (*tnf-α* and *il-1β*) in splenocytes exposed to LPS from fish fed the PPH diet. This is of special relevance since *tnf-α* is one of the earliest expressed immune genes during the infection process, having a key role in the activation of macrophages/phagocytes, as well as enhancing their antibacterial activity; thus, promoting leukocyte proliferation and migration [42,43]. Additionally, the other pro-inflammatory cytokine analysed in this study, IL-1β, is responsible for a cascade of effects on different members of this cytokine family, leading to signal transduction and activation of the nuclear factor (NF)-kB pathway, which regulates the inflammatory response, cellular growth, and apoptosis [44]. Although IL-1β is involved in the regulation of immunity through the stimulation of T cells [45], we did not find changes in gene expression of *cd4* mediated by the upregulation of this pro-inflammatory cytokine, which may be related to the complex regulation of the T cells’ differentiation by several cytokines and other transcription factors [46].

Anti-inflammatory cytokines may regulate the over-activation of immune responses and the further production of pro-inflammatory cytokines and other mediators; thus, mediating the balance between pro-inflammatory and anti-inflammatory responses [47], as well as preventing collateral damage to host tissues and avoid wasting bioenergetic resources [48]. Under the current ex vivo conditions, the expression levels of *il-10* and *tgf-β1* at 4 hpi were higher in splenocytes incubated with LPS from fish fed the PPH diet in comparison to the control group. Similar to other nutritional studies using in vivo and ex vivo models for evaluating the functional properties of plasma proteins in livestock [49,50,51] and mice [52], the upregulation of anti-inflammatory cytokines measured in this study, *il-10* and *tgf-β1*, confirmed the anti-inflammatory properties of the plasma proteins included in the PPH in front of the initial acute inflammatory response. This supports the hypotheses that plasma proteins (i.e., SDP) reduce over-stimulation of the mucosal immune response by enhancing IL-10 secretion and, therefore, allowing more of the available energy and nutrients to be used for growth and other productive functions rather than being diverted to the immune response [50].

Oxidative stress is generated from the imbalance between pro- and anti-oxidants in favour of the former, leading to the generation of oxidative damage. Part of the immune response relies on immune cells that eliminate pathogens by producing reactive oxygen species after immune stimulation [53]. Under the current experimental conditions and regardless of the experimental group considered (control and PPH diets), splenocytes stimulated with LPS did not show significant changes in *mn-sod* and *cat* expression in comparison to those incubated with PBS. These results are in disagreement with the response found in the intestine in a previous in vivo study where gilthead sea bream juveniles were fed plasma proteins (SDP) in diets with high FM levels and showed lower values of antioxidative stress activity [19]. However, results from both studies are not directly comparable due to the different plasma proteins evaluated, the diet formulation, and the experimental approach conducted for evaluating the oxidative stress condition.

The skin mucosa of fish is an essential barrier and serves as a protection against the surrounding environment. Considering that fish skin mucus provides a stable physical, biological, and chemical barrier against pathogens, proper knowledge of its antibacterial capacity when exposed to pathogenic organisms is of relevance, especially when evaluating the immune competence of fish [27,54]. Thus, in the present study, the authors evaluated some primary biomarkers of the skin mucus and ran several co-cultures of the skin mucus with non-pathogenic and pathogenic bacteria in order to evaluate the antibacterial capacity of the skin mucus in response to dietary conditions. The levels of glucose, lactate, cortisol, protein, and FRAP in the skin mucus samples and their ratios were not affected by the dietary condition. These parameters have been used for evaluating fish condition under different rearing situations, including biotic and abiotic stressors [24,26,55,56] and nutritional conditions [11,57]. Thus, the absence of differences in the chemical biomarkers of the skin mucus between both dietary groups indicated that the replacement of FM by PPH did not induce the stress response of gilthead seabream juveniles.

Regarding the co-culture of different bacteria with skin mucus from fish fed both experimental diets, in order to evaluate its inhibitory bacterial growth, the pathogenic bacteria chosen were *V. anguillarum* and *P. anguilliseptica*, which are reputed for being responsible for important diseases in gilthead sea bream [58]. In addition, a non-pathogenic bacterium, *E. coli*, was also chosen to evaluate whether skin mucus had a wider spectrum of activity regarding its antibacterial properties. Under current experimental conditions, the inclusion of PPH in diets formulated with low FM diets enhanced the antibacterial capacity of the skin mucus, as the co-culture of the three selected bacteria with skin mucus indicated. In addition to their role in either pathogen settlement or invasion of mucin carbohydrates produced by mucous cells, the skin mucus also serves as a repository of numerous innate immune components, such as glycoproteins, lysozyme, complement proteins, lectins, C-reactive protein, flavoenzymes, proteolytic enzymes, and antimicrobial peptides, as well as immunoglobulins, which exert inhibitory or lytic activity against pathogenic microorganisms [54]. Thus, the higher growth inhibitory capacity of skin mucus from fish fed the PPH when compared to the control group confirmed the immunomodulatory properties of the tested ingredient as indicated by the ex vivo trial, although we did not evaluate which mucus immune components were responsible for such antibacterial activity. The abovementioned results regarding the immunomodulatory capacity of the PPH on splenocytes exposed to bacterial LPS and the antibacterial activity of skin mucus co-cultured with different bacteria may be attributed to the bioactive compounds present in PPH, such as immunoglobulins, albumins, growth factors, and biologically active peptides, which may mediate the anti-inflammatory and immunomodulatory effects [38,59,60,61].

## 5. Conclusions

Results from the present study indicate that porcine protein hydrolysate obtained from blood plasma (PEPTEIVA^®^) is a safe and functional ingredient for aquafeeds, especially in those formulated diets with low FM levels. In particular, PPH promoted somatic growth and improved feed performance, whereas at the same time it enhanced the immune response in gilthead sea bream. The present results further indicate that the mode of action of PPH in terms of fish performance and immunity may be similar to the spray-dried plasma, the most common blood by-product. Considering these beneficial properties, this ingredient can be useful to incorporate into aquafeeds.

## Figures and Tables

**Table 1:** Ingredient list and proximate composition of the experimental diets evaluated in gilthead sea bream (*Sparus aurata*) juveniles.

Ingredients (%)	Control Diet	PPH Diet
Fishmeal LT70 (NORVIK)	7.0	2.0
PPH (PEPTEIVA^®^, APC Europe)	-	5.0
Soy protein concentrate (Soycomil)	21.0	21.0
Pea protein concentrate	12.0	12.0
Wheat gluten	12.0	12.0
Corn gluten	12.0	12.0
Soybean meal 48	5.0	5.0
Wheat meal	10.4	10.4
Fish oil (SAVINOR)	15.0	15.0
Vitamin and Mineral Premix PV01	1.0	1.0
Soy lecithin—Powder	1.0	1.0
Binder (guar gum)	1.0	1.0
MCP	2.0	2.0
L-Lysine	0.3	0.3
L-Tryptophan	0.1	0.1
DL-Methionine	0.2	0.2
**Proximate composition**		
Crude protein (%)	48.37 ± 0.2	48.50 ± 0.3
Crude fat (%)	17.19 ± 0.2	17.11 ± 0.1
Ash (%)	5.88 ± 0.05	5.81 ± 0.07
Gross energy (MJ kg feed^−1^)	21.62 ± 0.4	21.77 ± 0.5

**Table 2:** Key performance indicators of gilthead sea bream (*Sparus aurata*) juveniles fed low fishmeal diets containing 5% of porcine protein hydrolysate (PPH). Data are shown as the mean ± standard deviation. The asterisk denotes statistically significant differences between both experimental groups (*t*-test, *p* < 0.05).

	Control Diet	PPH Diet
Survival (%)	96.8 ± 2.7	97.6 ± 1.6
SL_i_ (cm)	10.2 ± 0.08	10.3 ± 0.10
SL_f_ (cm)	18.7 ± 0.31	19.0 ± 0.36
BW_i_ (g)	26.0 ± 0.03	25.9 ± 0.11
BW_f_ (g)	173.8 ± 4.14 *	182.2 ± 4.37
SGR_BW_ (% BW day^−1^)	2.06 ± 0.03 *	2.12 ± 0.02
K	2.64 ± 0.07	2.67 ± 0.04
FCR	0.87 ± 0.06	0.93 ± 0.11
FI (g feed fish^−1^)	115.1 ± 4.5 *	130.8 ± 8.7

Abbreviations: SL_i_, initial standard length; SL_f_, final standard length; BW_i_, initial body weight; BW_f_, final body weight; SGRBW, specific growth rate in terms of BW; K, Fulton’s condition factor; FCR, feed conversion ratio; FI, feed intake.

**Table 3:** Skin mucus biomarkers and their ratios from gilthead sea bream (*Sparus aurata*) juveniles fed low fishmeal diets containing 5% of porcine protein hydrolysate (PPH). Values are mean ± standard deviation from ten individual fish per dietary condition.

	Control Diet	PPH Diet
**Mucus biomarkers**		
Glucose (µg mL^−1^)	14.98 ± 5.45	16.36 ± 5.61
Lactate (µg mL^−1^)	7.24 ± 3.01	8.57 ± 2.37
Protein (mg mL^−1^)	9.01 ± 3.41	11.76 ± 3.46
Cortisol (µg mL^−1^)	0.40 ± 0.45	0.06 ± 0.02
FRAP (µmol mL^−1^)	1387 ± 277	1610 ± 294
**Mucus ratios**		
Glucose/protein (µg mg^−1^)	1.83 ± 0.53	1.66 ± 0.49
Lactate/protein (µg mg^−1^)	0.82 ± 0.17	0.76 ± 0.18
Cortisol/protein (ng g^−1^)	53.4 ± 57.6	7.2 ± 3.3
FRAP/protein (µmol mg^−1^)	146 ± 20	146 ± 41
Glucose/Lactate	2.41 ± 0.44	2.02 ± 0.46

## Data Availability

The data that support the findings presented in this study are available in “Repositorio IRTA” upon direct request to the corresponding author.
